# Supplementary material for: Comparison of the Aroma-Active Compounds and Sensory Characteristics of Different Grades of Light-Flavor Baijiu
Source: Foods. 2023 Mar 14;12(6):1238. doi: 10.3390/foods12061238 (PMC10048497; doi:10.3390/foods12061238)
Supplement: Supplementary file 1 [file foods-12-01238-s001.zip › foods-2254320-supplementary.pdf]

Table S1 . Definition and reference of the retronasal aroma attributes

No.	Retronasal aroma attributes	Definition	reference
1	Alcoholic	The aroma of ethanol	50% aqueous ethanol
2	Grain	The aroma of fermented grains	5mL of sorghum juice
3	Acidic	The aroma of volatile acids	10% aqueous ethanol solution of 1g/L acetic acid
4	Fruity	The aroma of ripe fruit	10% aqueous ethanol solution of 50mg/L ethyl hexanoate, 2g/L ethyl acetate, 6mg/L isoamyl acetate
5	Floral	The aroma of fresh flowers	10% aqueous ethanol solution of 50mg/L 2-phenylethanol, 5mg/L phenethyl acetate
6	Roasted	The aroma similar to fried rice	10% aqueous ethanol solution of 1mg/L Trimethylpyrazine
7	Grassy	The aroma of grass	10% aqueous ethanol solution of 5mg/L hexanal
8	Earthy	The aroma of fresh earthy	10% aqueous ethanol solution of 0.2μg/L geosmin

Note: Before the formal experiment, 2 sessions were used to deepen the panelists' perception of these 8 attributes. Panelists tasted the reference samples, except acidic (too strong taste) and earthy (no food-grade standard), to perceive different retronasal aroma attributes. The references of acidic and earthy only need to smell.

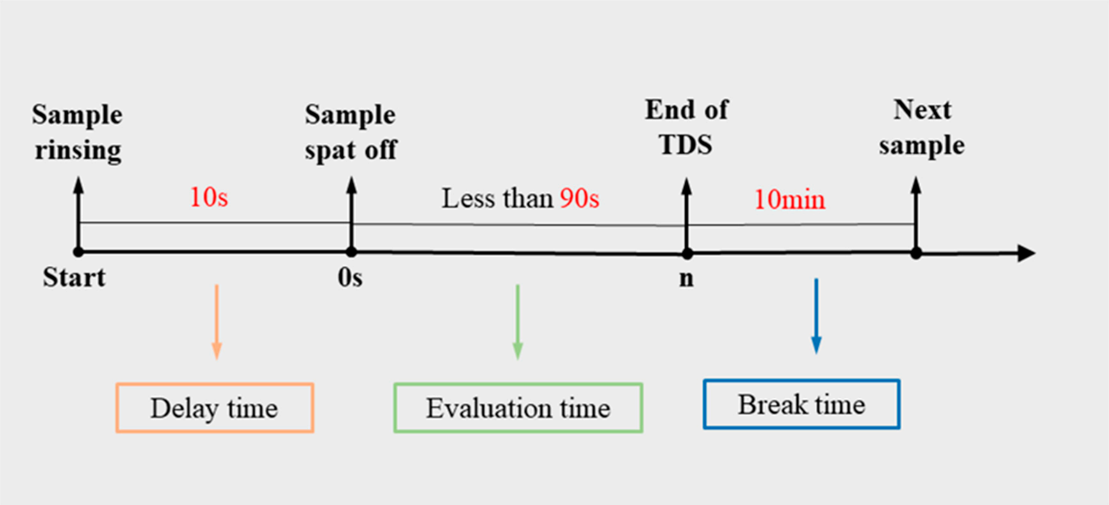

Figure S1. The evaluation procedure of TDS

Table S2 . Definition and reference of the orthonasal aroma attributes

No.	Orthonasal aroma attributes	Definition	Reference	Score
1	Grain	The aroma of fermented grains	5 g steamed sorghum	7
2	Alcoholic	The aroma of ethanol	50% aqueous ethanol	7
3	Floral	The aroma of fresh flowers	50% aqueous ethanol solution of 10mg/L 2-phenylethanol and 5mg/L 2-phenylethyl acetate	7
4	Fruity	The aroma of ripe fruit	50% aqueous ethanol solution of 10mg/L ethyl hexanoate, 2g/L ethyl acetate, 6mg/L 3-methylbutyl acetate	7
5	Grassy	The aroma of grass	50% aqueous ethanol solution of 2mg/L hexanal	7
6	Acidic	The aroma of volatile acids	50% aqueous ethanol solution of 1g/L acetic acid	7
7	Sweet	The aroma of honey	50% aqueous ethanol solution of 100 μg/L β-damascenone	7

Table S3 . QDA scoring standard

Score	Standard
0	The aroma is not perceived at all
1	The aroma is very weak, almost none
2	The aroma can be perceived, but the intensity is very weak
3	The aroma can be perceived, but the intensity is weak
4	The aroma can be clearly perceived, the intensity is medium
5	The aroma can be clearly perceived,and the intensity is strong
6	The intensity of aroma is strong, but lower than the reference
7	The intensity of the aroma is similar to the reference

Table S4. Quantitative methodological parameters of aroma compounds in four different grades of FJ.

No.	Aroma compound	IS [a]	Quantitative Ion (m/z)	Calibration curve			Range (μg/L)	LOD [b] (μg/L)	LOQ [c] (μg/L)	Recovery (%)
				Slope	Intercept	R^2				
1	2-Methylpropanal	IS3	72	24.037	0.9425	0.9949	29.02~3714.04	31.09	103.63	87.15
2	1,1-Diethoxyethane[d]	IS2	-	0.5573	0.0471	0.9971	15830~506680	186.28	620.93	93.74
3	3-Methylbutanal	IS3	58	37.531	6.2899	0.9979	181.75~46527.04	57.39	191.31	117.67
4	Ethyl acetate[d]	IS2	-	1.1173	0.1405	0.9993	66440~2126140	664.42	2214.73	84.65
5	Ethyl propanoate	IS4	57	303.2	4.9645	0.9938	156.25~80001.91	234.38	781.27	90.48

6	Ethyl 2-methylpropanoate	IS4	71	87.562	2.9627	0.9904	57.73~14780	26.24	87.48	86.92
7	2-Methylpropyl acetate	IS4	56	71.768	1.7851	0.9975	32.83~16810	3.22	10.73	116.20
9	Ethyl butanoate	IS4	71	49.301	1.7781	0.9983	60.37~30910	23.74	79.14	89.20
10	1-Propanol[d]	IS1	-	0.9921	0.006	0.9995	26590~851030	265.95	886.49	101.22
11	Ethyl 2-methylbutanoate	IS4	102	24.136	0.2673	0.9967	5.63~2885	2.82	9.39	105.34
13	Ethyl 3-methylbutanoate	IS4	88	18.398	0.3021	0.9967	6.65~3406	6.05	20.16	118.31
14	Hexanal	IS3	56	8.3514	-0.2711	0.9987	6.75~6913.45	4.31	14.36	104.09
15	2-Methylpropanol[d]	IS1	-	0.854	0.0053	0.9995	9380~300010	110.30	367.66	83.13
16	1-Butanol	IS3	56	111.35	-3.6178	0.9969	315.7~10102.26	295.96	986.55	105.42
17	3-Methylbutyl acetate	IS4	70	41.13	-4.2469	0.9965	105.59~54061	1.04	3.46	106.80
18	Ethyl pentanoate	IS4	57	49.72	-0.0317	0.9986	9.99~5114	0.41	1.38	89.34
19	2-Heptanone	IS3	58	0.8688	-0.0239	0.9932	1.55~792.4	1.22	4.07	110.83
20	2-Pentylfuran	IS3	81	0.3848	0.2014	0.9944	13.43~3437.91	4.68	15.62	106.06
21	3-Methylbutanol[d]	IS1	-	0.7092	0.0084	0.9994	13460~430670	96.13	320.44	97.69
22	Ethyl hexanoate	IS4	88	14.936	0.2718	0.9924	161.45~20665.26	19.14	63.81	103.32
23	1-Pentanol	IS3	42	40.198	-1.0952	0.9981	31.05~7948.96	7.39	24.64	91.19
24	Hexyl acetate	IS4	56	11.058	-0.0016	0.9965	0.85~54.46	0.33	1.11	91.74
25	3-Methylbutyl butanoate	IS4	71	3.2413	-0.0005	0.9973	0.67~171.44	0.08	0.27	115.20
27	2-Heptanol	IS3	45	1.4178	0.1638	0.9928	3.48~1783.16	0.80	2.66	103.68
28	Ethyl heptanoate	IS4	88	4.2755	0.1113	0.9961	6.4~818.81	5.33	17.77	96.07
30	Ethyl lactate[d]	IS2	-	1.1307	0.0689	0.9966	71690~2293990	716.87	2389.57	105.45
31	1-Hexanol	IS3	55	25.427	-2.6501	0.9965	193.36~99000	25.78	85.94	100.20
32	2-Nonanone	IS3	58	0.2855	-0.066	0.997	1.26~646.87	0.37	1.23	109.41
34	Dimethyl trisulfide	IS3	126	0.8317	0.261	0.9912	1.46~746.85	0.54	1.80	113.28
35	Nonanal	IS3	98	2.2655	-0.1561	0.9969	6.79~1738	0.27	0.90	92.11
37	Acetic acid[e]	IS5	60	67.952	5.5823	0.9997	131522.81~33669840	1541.28	5137.61	110.11
38	Ethyl octanoate	IS4	88	4.5887	-1.9988	0.9982	100.36~12846	71.69	238.95	106.96
39	1-Heptanol	IS3	70	3.4364	-0.0214	0.9972	13.92~7126	10.99	36.63	92.08
40	3-Methylbutyl hexanoate	IS4	70	1.103	-0.0067	0.9997	0.67~342.68	0.24	0.79	95.72
41	Octyl acetate	IS4	70	5.6497	0.0015	0.9969	0.15~76.73	0.35	1.15	116.93
43	1-Octen-3-ol	IS3	57	1.0232	0.0277	0.9961	3.39~1734	0.19	0.64	85.78
44	Furfural	IS3	96	59.6	8.2453	0.9922	598.83~306600	105.06	350.19	96.80
46	Decanal	IS3	57	0.7702	-0.0567	0.9992	0.63~321.45	0.65	2.16	106.20
47	Benzaldehyde	IS3	106	1.3913	-0.4087	0.9942	5.86~749.87	0.16	0.52	116.56
48	Ethyl nonanoate	IS4	88	2.1613	-0.1251	0.999	6.82~3491.54	3.79	12.63	94.27
49	Propanoic acid[e]	IS5	74	11.218	0.2739	0.9995	1593.44~407919.8	810.22	2700.74	115.36
50	1-Octanol	IS3	56	2.0953	-1.4038	0.9981	19.75~10110	2.78	9.27	107.65
51	2-Methylpropanoic acid[e]	IS5	73	5.0427	0.0462	0.9948	531.17~33994.73	212.47	708.22	114.19

56	Butanoic acid[e]	IS5	60	2.3699	0.1592	0.9959	1594.72~51031.05	94.92	316.41	87.42
57	Ethyl decanoate	IS4	88	3.0516	-5.7189	0.9916	59.11~30264	1.57	5.23	86.70
58	1-Nonanol	IS3	56	0.9732	-1.421	0.9976	14.88~7616.96	1.26	4.21	116.94
59	Phenylacetaldehyde	IS3	91	7.5237	0.7493	0.9978	7.79~31917	7.54	25.14	93.34
60	Ethyl benzoate	IS4	105	5.0435	-0.1309	0.995	3.09~1582.62	0.22	0.72	111.21
62	2-Methylbutanoic acid[e]	IS5	74	2.0487	0.0155	0.9992	265.89~17017.24	35.14	117.13	114.11
63	3-Methylbutanoic acid[e]	IS5	60	1.2193	0.0474	0.9978	532.53~34082.18	29.05	96.82	120.76
64	Diethyl butanedioate	IS4	101	145.25	-1.4796	0.9941	626.07~320548.23	16.01	53.37	117.40
67	Pentanoic acid[e]	IS5	60	1.4811	0.0573	0.999	543.73~34798.74	56.25	187.49	89.89
68	Naphthalene	IS3	128	0.0402	0.017	0.9961	1.57~200.39	0.90	3.01	109.72
70	Ethyl phenylacetate	IS4	91	5.289	0.0025	0.9949	1.5~383.92	0.11	0.36	104.11
71	2-Phenylethyl acetate	IS4	104	6.0501	0.1366	0.9934	3.1~1587.5	0.32	1.07	112.83
72	β-Damascenone	IS3	69	0.5911	-0.1356	0.9997	1.56~3188.48	0.85	2.83	112.13
73	Ethyl dodecanoate	IS4	88	1.755	-0.0538	0.998	8.64~4425	5.76	19.21	98.98
74	Hexanoic acid[e]	IS5	60	2.1153	0.189	0.995	1586.55~101538.99	30.45	101.51	104.03
76	Geranylacetone	IS3	69	0.1756	-0.0226	0.9999	0.16~673	0.02	0.05	97.15
77	Guaiacol	IS3	109	15.645	-0.1192	0.9972	1.93~493.75	0.70	2.32	108.97
78	Ethyl 3-phenylpropanoate	IS4	104	3.5418	0.0778	0.9956	1.79~917.94	0.16	0.54	86.78
79	2-Phenylethanol	IS3	91	18.201	-2.1329	0.9956	98.44~50400	8.63	28.78	104.86
80	β-Ionone	IS3	177	0.1662	0.0071	0.9982	0.04~151	0.02	0.06	89.71
81	4-Methylguaiacol	IS3	123	5.0918	-0.0139	0.9989	2.35~601.6	4.41	14.69	109.44
82	Phenol	IS3	94	164.98	-0.9879	0.9977	16.38~8385.6	8.62	28.73	98.48
84	4-Ethylguaiacol	IS3	137	1.1941	0.0709	0.9982	1.94~1983	0.29	0.97	103.62
85	Ethyl tetradecanoate	IS4	88	1.7726	0.0619	0.9909	0.62~319.56	1.34	4.46	88.71
86	Octanoic acid[e]	IS5	60	1.9688	0.0893	0.995	538.51~34464.84	74.79	249.31	106.68
87	4-Ethylphenol	IS3	107	3.5258	-0.4433	0.9984	6.22~3182.19	2.78	9.28	104.29
88	Decanoic acid[e]	IS5	60	0.4085	0.057	0.9907	425.14~13604.36	102.86	342.85	99.42

[a] IS, internal standard; IS1, 2-methyl-2-butanol; IS2, n-pentyl acetate; IS3, dl-mentho; IS4, octyl propanoate; IS5, trimethylacetic acid. [b] LOD, limit of detection, the analyte concentration of a standard that produced a signal-to-noise ratio of 3. [c] LOQ,limit of quantitation, the analyte concentration of a standard that produced a signal-to-noise ratio of 10. [d] These compounds were quantitated by GC-FID method. [e] These compounds were quantitated by LLME coupled with GC-MS method. Other compounds were quantified by HS-SPME-GC-MS method.

(A)

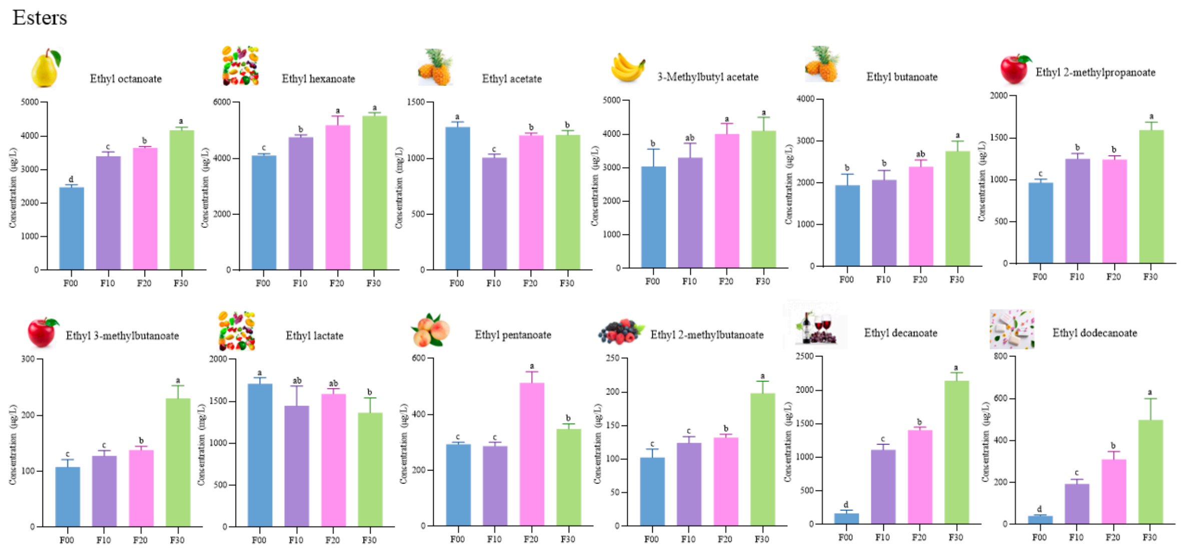
Esters
Ethyl octanoate
Ethyl hexanoate
Ethyl acetate
3-Methylbutyl acetate
Ethyl butanoate
Ethyl 2-methylpropanoate
Ethyl 3-methylbutanoate
Ethyl lactate
Ethyl pentanoate
Ethyl 2-methylbutanoate
Ethyl decanoate
Ethyl dodecanoate
Concentration (μg/L)
F00
F10
F20
F30

(B)

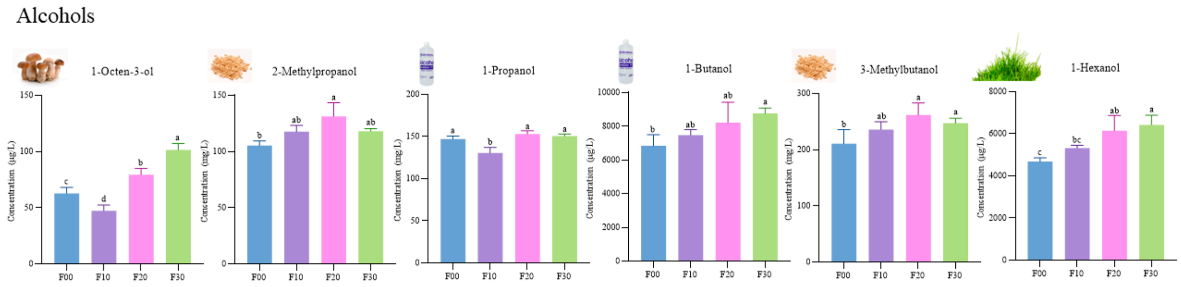
Alcohols
1-Octen-3-ol
2-Methylpropanol
1-Propanol
1-Butanol
3-Methylbutanol
1-Hexanol
Concentration (μg/L)
F00
F10
F20
F30

(C)

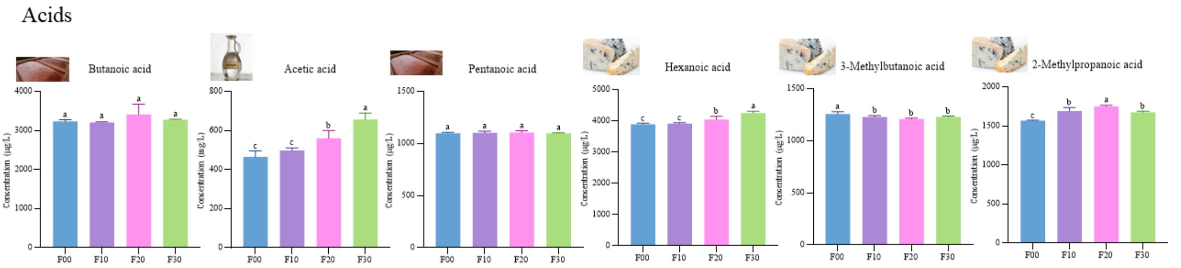
Acids
Butanoic acid
Acetic acid
Pentanoic acid
Hexanoic acid
3-Methylbutanoic acid
2-Methylpropanoic acid
Concentration (μg/L)
F00
F10
F20
F30

(D)

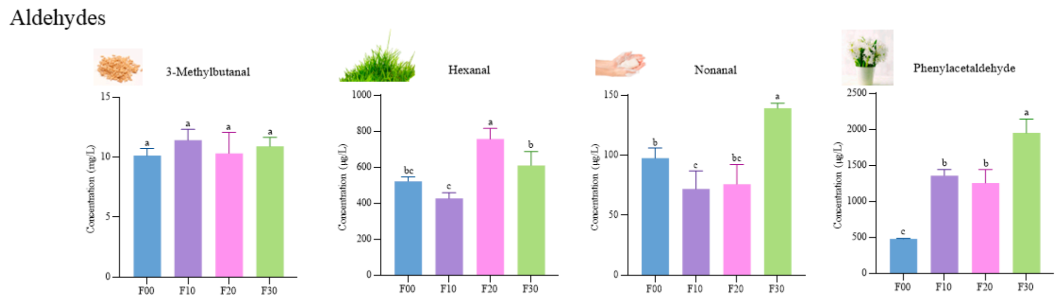
Aldehydes
3-Methylbutanal
Hexanal
Nonanal
Phenylacetaldehyde
Concentration (μg/L)
F00
F10
F20
F30

(E)

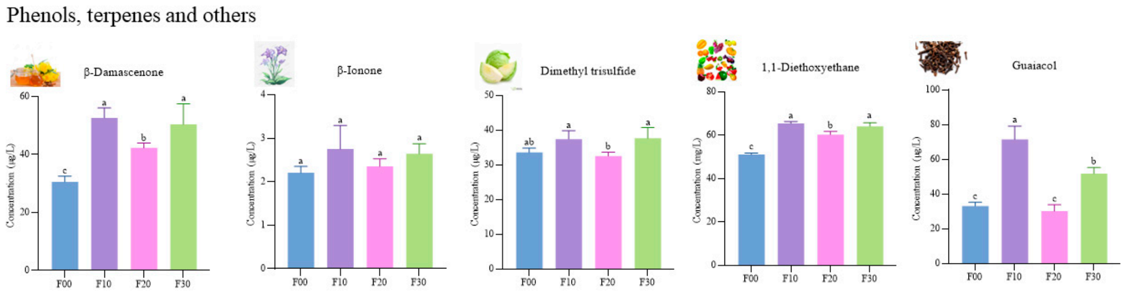

Figure S2. Concentrations of aroma-active compounds with OAVs＞1 in four different grades of FJ. (A) Esters with OAVs＞1 in F30, (B) Alcohols, (C) Acids, (D) Aldehydes, (E) Phenols, terpenes and others. Note: The concentrations of 3-methylbutanal, ethyl acetate, 1,1-diethoxyethane, ethyl lactate, 2-methylpropanol, acetic acid, 1-propanol and 3-methylbutanol were mg/L.
